# Regenerative potential of induced pluripotent stem cells derived from patients undergoing haemodialysis in kidney regeneration

**DOI:** 10.1038/s41598-018-33256-7

**Published:** 2018-10-08

**Authors:** Susumu Tajiri, Shuichiro Yamanaka, Toshinari Fujimoto, Kei Matsumoto, Atsuhiro Taguchi, Ryuichi Nishinakamura, Hirotaka James Okano, Takashi Yokoo

**Affiliations:** 1 Division of Nephrology and Hypertension, Department of Internal Medicine, The Jikei University School of Medicine, 3-25-8, Nishi-Shimbashi, Minato-ku, Tokyo 105-8461 Japan; 2 Department of Kidney Development, Institute of Molecular Embryology and Genetics, Kumamoto University, 2-2-1, Honjo, Chuo-ku, Kumamoto, 860-0811 Japan; 3 Department of Genome Regulation, Max Planck Institute for Molecular Genetics, Ihnestraße 63-73, 14195 Berlin, Germany; 4 Division of Regenerative Medicine, The Jikei University School of Medicine, 3-25-8, Nishi-Shimbashi, Minato-ku, Tokyo 105-8461 Japan

## Abstract

Kidney regeneration from pluripotent stem cells is receiving a lot of attention because limited treatments are currently available for chronic kidney disease (CKD). It has been shown that uremic state in CKD is toxic to somatic stem/progenitor cells, such as endothelial progenitor and mesenchymal stem cells, affecting their differentiation and angiogenic potential. Recent studies reported that specific abnormalities caused by the non-inherited disease are often retained in induced pluripotent stem cell (iPSC)-derived products obtained from patients. Thus, it is indispensable to first assess whether iPSCs derived from patients with CKD due to non-inherited disease (CKD-iPSCs) have the ability to generate kidneys. In this study, we generated iPSCs from patients undergoing haemodialysis due to diabetes nephropathy and glomerulonephritis (HD-iPSCs) as representatives of CKD-iPSCs or from healthy controls (HC-iPSCs). HD-iPSCs differentiated into nephron progenitor cells (NPCs) with similar efficiency to HC-iPSCs. Additionally, HD-iPSC-derived NPCs expressed comparable levels of NPC markers and differentiated into vascularised glomeruli upon transplantation into mice, as HC-iPSC-derived NPCs. Our results indicate the potential of HD-iPSCs as a feasible cell source for kidney regeneration. This is the first study paving the way for CKD patient-stem cell-derived kidney regeneration, emphasising the potential of CKD-iPSCs.

## Introduction

Chronic kidney disease (CKD) is a major problem worldwide and the number of patients with CKD continues to rise^1,2^. The replacement of kidney function in patients with end-stage renal disease requires dialysis or kidney transplantation. Although kidney transplantation can improve the quality of life and prolong the life expectancy of patients with CKD^3^, the insufficient number of donor organs make this a suboptimal solution in the treatment of severe renal diseases^4^. Hence, kidney regeneration by induced pluripotent stem cells (iPSCs) is expected to be particularly helpful.

Kidneys arise from metanephros, which develop via the reciprocal interaction between the metanephric mesenchyme, containing nephron progenitor and stromal progenitor cells and the ureteric bud (UB)^5^. Recently, kidney regeneration from pluripotent stem cells (PSCs) has made remarkable progress and several studies have reported the successful differentiation of PSCs into nephron progenitor cells (NPCs) and UB *in vitro*, by recapitulating the development of the metanephric kidney^6–11^. However, because kidney development is a very complex process, we need to combine NPCs, stromal progenitor cells and UB spatiotemporally at an appropriate ratio for kidney generation. More importantly, stromal progenitor cells have not yet been generated from PSCs and it will still take time to generate whole kidneys from PSCs *in vitro*.

To generate whole functional kidneys, we have employed the ‘organogenic niche method’, which uses heterozygous embryos as an organ factory^12–14^. In this method, progenitor cells are applied at the area of nephrogenesis, where they differentiate into kidneys by borrowing the developing programs of the growing xeno-embryos. Notably, we have recently generated transgenic embryos conditionally expressing the diphtheria toxin receptor and treated them with diphtheria toxin to ablate native NPCs from the nephrogenic area. Then, we applied allogenic (mouse) and xenogenic (rat) exogenous NPCs to the nephrogenic area and succeeded in replacing native NPC with the exogenous NPCs in isolated mouse metanephros^15^. In this system, the native niche supports the differentiation of exogenous NPCs into neo-nephrons, while exogenous NPCs support the proliferation of native UB. Importantly, the distal neo-nephrons connected with the native UB. In such way, we have succeeded in providing a suitable niche for exogenous NPCs, while keeping the original structure of the metanephros.

iPSCs generated from patients are a promising tool among the tailor-made therapeutic approaches because they supply patient-derived cells/tissues/organs. The regeneration of kidneys derived from iPSCs of patients with CKD will help to circumvent current problems such as organ shortages^4^, immune rejection and life-long immunosuppression^16^. We plan to use NPCs differentiated from iPSCs derived from patients with CKD that is not due to a genetic cause (CKD-iPSCs) as a cell source for the strategy described above, after optimizing it for human cells^15^, to regenerate kidneys from CKD-iPSCs. Because non-inherited kidney diseases are the major cause of CKDs, such as diabetic nephropathy and glomerulonephritis, a considerable proportion of patients with CKD would be able to benefit from this procedure. Although the cell products derived from inherited kidney disease-specific iPSCs demonstrate a disease specific phenotype^17^, nothing has been reported on the biological properties of cell products derived from patients with CKD due to non-inherited causes. Recently, several studies have shown that non-inherited disease-specific abnormalities are often retained in iPSC-derived products obtained from patients^18–20^. CKD leads to accumulation of organic compounds (uremic toxins) in the bloodstream; these cause many toxic effects, including reduced proliferation capacity, differentiation abnormalities,^21,22^ and angiogenic function^22–27^ in stem and progenitor cells and premature aging in stem^27^ and peripheral blood mononuclear cells (PBMCs)^28^.

Hence, to generate CKD-iPSC-derived kidneys, it is indispensable to first assess whether the CKD-iPSCs and CKD-iPSC-derived products retain the adverse CKD-specific abnormalities, especially the differentiation ability and angiogenic function, which are crucial for kidney regeneration. Therefore, in this study, we generated iPSCs from haemodialysis (HD) patients with CKD that was not due to a genetic cause (HD-iPSCs) and used them as representatives of CKD-iPSCs; additionally, we also generated iPSCs from age- and sex-matched healthy controls (HC; HC-iPSCs). Then, we compared the characteristics of HD-iPSCs, HD-iPSC-derived NPCs and HD-iPSC-derived nephrons to those from HC-iPSCs. In such way, we assessed the potential of HD-iPSCs as cell source for kidney regeneration.

## Results

### Generation of iPSCs from patients on haemodialysis and healthy controls

**Figure 1:**
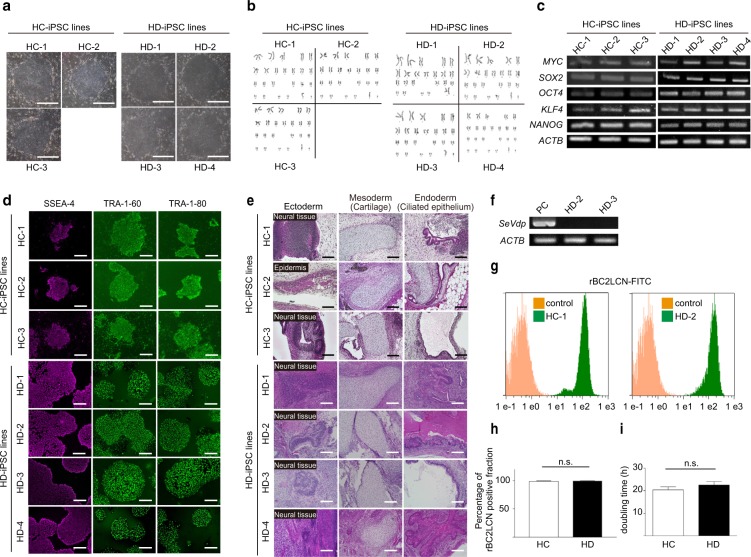
Characterisation of HC-iPSCs and HD-iPSCs. (**a**) Morphology of HC-iPSC and HD-iPSC colonies. Scale bars: 500 µm. (**b**) Karyotype of HC-iPSC and HD-iPSC lines. HC-1: 46, XY; HC-2: 46, XY; HC-3: 46, XY; HD-1: 46, XX; HD-2: 46, XY; HD-3: 46, XY; HD-4: 46, XY. (**c**) RT-PCR for stem cell markers of HC-iPSC and HD-iPSC lines. (**d**) Immunostaining of the stem cell markers in HC-iPSC lines (on feeder condition) and HD-iPSC lines (feeder-free condition). Scale bars, 100 µm. (**e**) HE-stained sections of teratoma derived from HC-iPSC and HD-iPSC lines. Scale bars, 100 µm. (**f**) Confirmation of the absence of SeVdp-related reprogramming gene by RT-PCR. PC, control transfected cell sample. (**g**) rBC2LCN positive cell analysis in the HC-1 and HD-2 by flow cytometry. (**h**) Comparison of the percentage of the rBC2LCN positive population between the HC- and HD-iPSC lines (n = 4 in each groups). (**i**) Comparison of the doubling time between the HC- and HD-iPSC lines (n = 4 in each group). Full-length gel is presented in Supplementary Figure S4. HC, healthy controls; HD, haemodialysis; SeVdp, Sendai virus vector. Data are the mean ± SEM (two-tailed, unpaired t-test).

**Figure 2:**
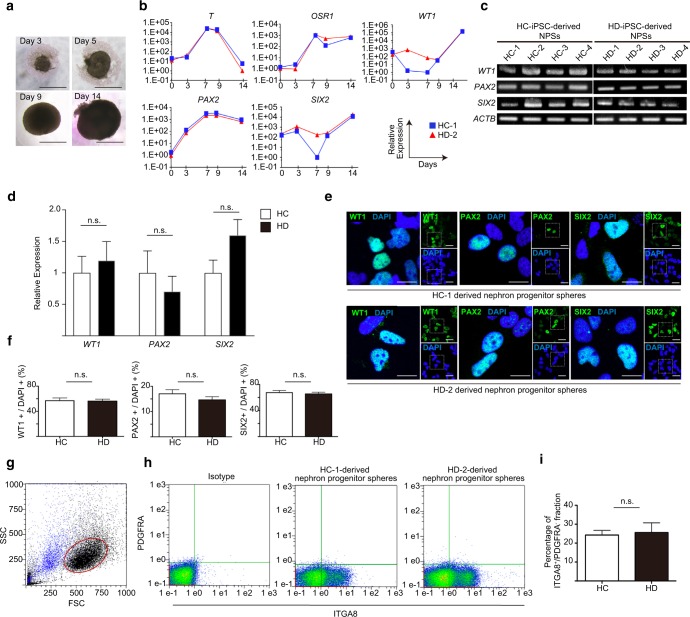
Comparison of the NPC induction efficiency between HC- and HD-iPSC lines. (**a**) The size of spheres derived from HD-2 increased over time. Scale bars: 500 µm. (**b**) qRT-PCR profiling of *T*, *OSR1*, *PAX2*, *WT1* and *SIX2* of the spheres derived from HC-1 and HD-2 at 5 points during induction from iPSCs to NPSs. (**c**) RT-PCR for NPC marker gene expression, *WT1*, *PAX2* and *SIX2*, of HC- and HD-iPSC-derived NPSs. (**d**) qRT-PCR relative to the expression of *WT1*, *PAX2* and *SIX2*, between the HC- and HD-iPSC-derived NPSs (n = 12 in each group). (**e**) Immunostaining of WT1, PAX2 and SIX2 in dissociated HC-1 and HD-2-derived NPSs. Scale bars, 20 µm. (**f**) Comparison of the percentage of the WT1, PAX2 and SIX2 positive cells between the HC- and HD-iPSC-derived NPSs (n = 12 in each group). (**g**) Forward and side scatter plots of dissociated NPSs. Blue: DAPI positive dead cells. (**h**) Flow cytometric analysis of the HC-1 and HD-2-derived NPSs in the gate. (**i**) Comparison of the percentage of the ITGA8^+^/PDGFRA^−^ population between the HC- and HD-iPSC-derived NPSs (n = 4 in each group). Full-length gel is presented in Supplementary Figure S5. NPCs, nephron progenitor cells; NPSs, nephron progenitor spheres; HC, healthy controls; HD, haemodialysis. Data are the mean ± SEM (two-tailed, unpaired t-test).

Three patients with end-stage renal disease attending our institution for HD were enrolled in the study. All HD patients had received standard dialysis therapy for renal insufficiency. Of the three patients, one had chronic glomerulonephritis, one had diabetic nephropathy and one had rapidly progressive glomerulonephritis. HD-iPSC lines were generated from PBMCs of these patients. Similar to HD-iPSC lines, HC-iPSC lines were generated from PBMCs of two healthy controls. iPSC donor characteristics are presented in Table 1. Although the iPSC generation efficiency varied among the individuals, we succeeded in generating four HD-iPSC lines, namely HD-1, HD-2, HD-3 and HD-4 and three HC-iPSC lines, namely HC-1, HC-2 and HC-3. All iPSC lines used in this study showed the typical colony morphology of iPSCs^29^ (Fig. 1a), maintained a normal karyotype (Fig. 1b) and met the authenticity criteria of iPSCs: (1) expression of stem cell markers (Fig. 1c); (2) expression of human embryonic stem cell (hESC)-specific surface antigens, including stage-specific embryonic antigen-4 (SSEA-4), tumour-related antigen (TRA)-1-60 and TRA-1-81 (Fig. 1d); and (3) ability to differentiate into the three germ layers through teratoma formation (Fig. 1e). In HD-2 and HD-3, the replication-defective and persistent Sendai virus (SeVdp) vector was completely removed by RNA interference (Fig. 1f). Additionally, we also used 201B7 cells obtained from the RIKEN BioResource Centre as HC-iPSCs, namely HC-4, for matching patient age and sex against HD-iPSC lines. Flow cytometric analysis using rBC2LCN, which is a novel detection reagent for iPSCs^30^, showed that almost all the cells were rBC2LCN positive and there was no significant difference between the two groups (HC and HD group, n = 4 in each, Fig. 1g,h, Supplementary Figure S1). We cultured the iPSCs on feeders at first and then transitioned them to feeder-free conditions for differentiation. All iPSC lines reached 80–90% confluence under feeder-free conditions in 6-well plates after approximately 7–8 days. We found no significant differences in doubling time between the two groups (HC and HD group, n = 4 in each, Fig. 1i).

**Table 1:** iPSC lines derived from healthy controls and patients on haemodialysis.

	Healthy controls (HC)			Haemodialysis patients (HD)		
Identifier in the study	HC-1, 2	HC-3	HC-4	HD-1	HD-2, 3	HD-4
iPSC line No.	1401#7, #15	1406 #22	201B7	HD1 #11	HD2 #6, #7	HD5 #6
Age^a^	60 s	50 s	36	39	65	50
Sex	Male	Male	Female	Female	Male	Male
Duration of RRT (months)				46	57	85
Cause of CKD				CGN	DMN	RPGN
Cell source	PBMCs	PBMCs	Fibroblasts	PBMCs	PBMCs	PBMCs
Vector	Episomal plasmid	Episomal plasmid	Retrovirus	Episomal plasmid	Sendaivirus	Episomal plasmid
Reprogramming factors	*SOX2*, *OCT3/4*, *KLF4*, *L-MYC*, *LIN28*, p53-shRNA	*SOX2*, *OCT3/4*, *KLF4*, *L-MYC*, *LIN28*, p53-shRNA	*SOX2*, *OCT3/4*, *KLF4*, *c-MYC*	*SOX2*, *OCT3/4*, *KLF4*, *L-MYC*, *LIN28*, p53-shRNA	*SOX2*, *OCT3/4*, *KLF4*, *c-MYC*	*SOX2*, *OCT3/4*, *KLF4*, *L-MYC*, *LIN28*, p53-shRNA

Abbreviations: CKD, chronic kidney disease; CGN, chronic glomerulonephritis; DMN, diabetic nephropathy; PBMCs, peripheral blood mononuclear cells; RPGN, rapidly progressive glomerulonephritis; RRT, renal replacement therapy; ^a^The exact ages of the healthy control subjects, i.e., HC-1, HC-2 and HC-3 are unknown because of unlinkable anonymization.

### HD-iPSCs differentiated into NPCs as efficiently as HC-iPSCs

The four HD-iPSC lines and the four HC-iPSC lines were induced into nephron progenitor spheres (NPSs) including NPCs, using an embryoid body-mediated differentiation protocol^6^. The size of the spheres derived from the HD-iPSC lines increased over time (Fig. 2a). Quantitative reverse transcription polymerase chain reaction (qRT-PCR) showed that the spheres expressed specific markers such as Brachyury (*T*), Odd-skipped related transcription factor 1 (*OSR1*), Wilms tumour 1 (*WT1*), Paired box 2 (*PAX2*) and SIX homeobox 2 (*SIX2*) at the expected time points following the development of metanephric nephron progenitors^6^, similar to the HC-iPSC-derived spheres (Fig. 2b). On day 14, the NPSs, derived from both iPSC lines, expressed the NPC markers *WT1*, *PAX2* and *SIX2* (Fig. 2c).

We performed the following experiments to examine the NPC differentiation ability of HC- and HD-iPSC lines: (1) NPC marker expression analysis by qRT-PCR; (2) NPC marker protein expression analysis by immunostaining; and (3) NPC surface marker analysis by flow cytometry. For qRT-PCR, 6 NPSs were combined to represent one group and three groups for each iPSC line were analysed. We found no significant differences in the expression of the NPC markers *WT1*, *PAX2* and *SIX2*, between HC and HD groups (n = 12 in each group, Fig. 2d). For immunostaining, 12 NPSs were combined for each iPSC line. Again, we found no significant differences in the percentage of cells expressing NPC markers (Fig. 2e,f). For flow cytometry and cell sorting, 48 NPSs were combined for each iPSC line. We plotted forward versus side scatter of dissociated NPSs; DAPI positive dead cells are shown in blue (Fig. 2g). To eliminate cell debris and dead cells from the analysis, we placed the gate shown in red in the figure. Kaku *et al*.^31^ have shown that NPCs constitute an integrin subunit alpha 8 (ITGA8)^+^/platelet derived growth factor receptor alpha (PDGFRA)^−^ population. Therefore, we examined the ITGA8^+^/PDGFRA^−^ population in the gated cells and found no significant difference between HC- and HD-iPSC-derived NPSs (n = 4 in each group, Fig. 2h,i, Supplementary Figure S2).

### Isolated HC- and HD-iPSC-derived NPCs showed comparable levels of the nephron progenitor markers

**Figure 3:**
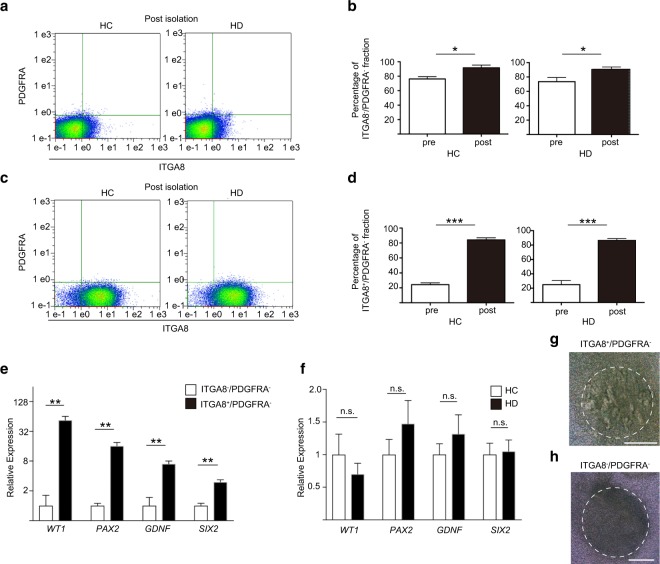
NPCs sorting from NPSs and comparison of the nephron progenitor marker expression levels in HC- and HD-iPSC-derived NPCs. (**a**) Flow cytometric analysis after PDGFRA negative and ITGA8 negative selection of HC- and HD-iPSC-derived NPSs. (**b**) Percentages of the ITGA8^−^/PDGFRA^−^ population in HC- and HD-iPSC-derived NPSs, pre and post sorting. (n = 4 in each group) (**c**) Flow cytometric analysis after PDGFRA negative and ITGA8 positive selection of HC- and HD-iPSC-derived NPSs. (**d**) Percentages of the ITGA8^+^/PDGFRA^−^ population in HC- and HD-iPSC-derived NPSs, pre and post sorting. (n = 4 in each group) (**e**) qRT-PCR relative to the level of expression of *WT1*, *PAX2*, *GDNF* and *SIX2*, between the ITGA8^−^/PDGFRA^−^ and the ITGA8^+^/PDGFRA^−^ population. (ITGA8^+^/PDGFRA^−^ population; n = 8, ITGA8^−^/PDGFRA^−^ population; n = 4). (**f**) qRT-PCR relative to the level of expression of *WT1*, *PAX2*, *GDNF* and *SIX2* in the post isolated ITGA8^+^/PDGFRA^−^ population between the HC and HD groups. (n = 4 in each group). (**g**,**h**) Isolated ITGA8 + /PDGFRA- aggregates showed tubulogenesis (**g**), while ITGA8-/PDGFRA- aggregates did not (**h**). NPCs, nephron progenitor cells; NPSs, nephron progenitor spheres; HC, healthy controls; HD, haemodialysis. Data are the mean ± SEM (two-tailed, unpaired t-test). *P < 0.05; **P < 0.01; ***P < 0.001.

To examine the iPSC-derived NPC characteristics between the HC and HD groups, we sorted NPCs from NPSs using the immunomagnetic separation method^32^. This system allows the separation of cells with lower damage and in shorter time when compared with conventional cell sorting methods. We isolated ITGA8^+^/PDGFRA^−^ cells in two steps. First, we performed a PDGFRA negative selection and then an ITGA8 positive selection (Supplementary Figure S3). In agreement with previous studies^6,31^, our results showed that the size of the PDGFRA^+^ population obtained using the embryoid body-mediated differentiation protocol was very limited. Next, we further separated, in this population, ITGA8 negative or positive cells. As a result, we successfully enriched both the ITGA8^−^/PDGFRA^−^ (from 76.2 ± 3.3% to 91.6 ± 3.8% in HC-iPSC-derived NPSs, p = 0.02 and from 74.2 ± 5.1% to 91.4 ± 2.6% in HD-iPSC-derived NPSs, p = 0.02, Fig. 3a,b, Supplementary Figure S2) and ITGA8^+^/PDGFRA^−^ (from 23.4 ± 3.2% to 84.3 ± 2.8% in HC-iPSC-derived NPSs, p < 0.0001 and from 25.6 ± 5.2% to 87.2 ± 1.9% in HD-iPSC-derived NPSs, p < 0.0001, Fig. 3c,d, Supplementary Figure S1) fractions. Then, we performed gene expression analyses of glial-cell derived neurotrophic factor (*GDNF*), which is one of the NPC markers and is a key signal of the interaction between NPCs and UB^5^, in addition to the analyses of *WT1*, *PAX2*, *SIX2*. The expression levels of NPC markers in the ITGA8^+^/PDGFRA^−^ population were higher than in the ITGA8^−^/PDGFRA^−^ population (ITGA8^+^/PDGFRA^−^ population; n = 8, ITGA8^−^/PDGFRA^−^ population; n = 4, Fig. 3e). We found no significant difference in the expression level of nephron progenitor markers in the ITGA8^+^/PDGFRA^−^ population between HC and HD groups (n = 4 in each group, Fig. 3f). Isolated ITGA8^+^/PDGFRA^−^ and ITGA8^−^/PDGFRA^−^ cells derived from HD-2 were cultured in 96-well plates for one day to form aggregates. Then, we co-cultured these aggregates with mouse embryonic spinal cords for nine days. ITGA8^+^/PDGFRA^−^ aggregates showed tubulogenesis (Fig. 3g), while ITGA8^−^/PDGFRA^−^ aggregates did not (Fig. 3h), as indicated in a previous study that used the conventional cell sorting method^31^. These data indicate that we could purify iPSC-derived NPCs using the immunomagnetic separation method and that the immunomagnets did not affect the differentiation of isolated iPSC-derived NPCs.

### HD-iPSC-derived NPCs showed possibility to differentiate into nephrons similar to the HC-iPSC-derived NPCs

**Figure 4:**
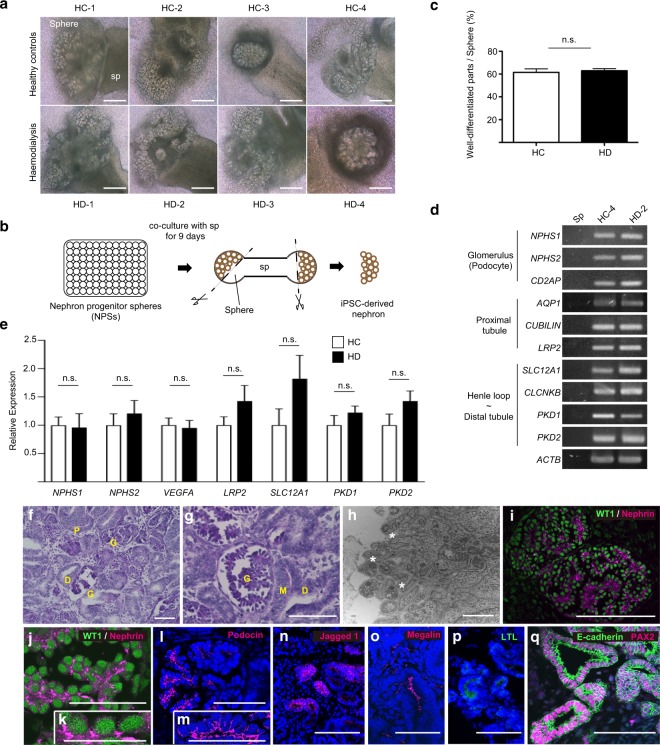
Gene expression and morphological and immunological analyses of the HC- and HD-iPSC-derived nephrons. (**a**) NPSs, co-cultured with mouse embryonic spinal cords for nine days, showed robust tubulogenesis. Scale bars: 500 µm. (**b**) Schematic of the analysis of the gene expression in iPSC-derived nephrons. (**c**) Comparison of the percentage of the well-differentiated parts per sphere (used in gene expression assay, n = 12 in each group). (**d**) RT-PCR relative to the level of gene expression in multiple nephron segments including podocytes, proximal and distal tubules of HC-4-derived nephrons and HD-2-derived nephrons. Mouse embryonic spinal cord (Sp) was used as negative control. (**e**) qRT-PCR relative to the expression of the nephron markers *NPHS1*, *NPHS2*, *VEGFA*, *LRP2*, *SLC12A1*, *PKD1* and *PKD2* in HC- and HD-iPSC-derived nephrons (n = 12 in each group). (**f**,**g**) PAS-stained sections of HD-1-derived nephrons. G, glomerulus; P, proximal tubule; D, distal tubule; M, macula densa. Scale bars, 100 µm. (**h**) Transmission electron microscopic images of primary processes of induced glomeruli (asterisks). Scale bar, 500 nm. (**i**–**q**) Immunostaining for HD-1-derived glomerular markers (**i**–**m**), proximal tubule markers (**n**–**p**) and distal tubule markers (**q**). Scale bars, 50 µm (j-m) or 100 µm (**i**,**n**–**q**). Full-length gel is presented in Supplementary Figure S6. NPSs, nephron progenitor spheres; HC, healthy controls; HD, haemodialysis. Data are the mean ± SEM (two-tailed, unpaired t-test).

Next, we examined whether HD-iPSC-derived NPCs could differentiate into nephrons similar to HC-iPSC-derived NPCs. We co-cultured NPSs including NPCs with mouse embryonic spinal cords for nine days. Although the differentiation efficiency varied among NPSs, most NPSs underwent robust tubulogenesis (Fig. 4a). We selected three well-differentiated spheres from each iPSC line, separated the well-differentiated parts, named iPSC-derived nephrons and used them for further analysis (Fig. 4b). We found no significant difference in the percentage of iPSC-derived nephrons per sphere between the HC and HD groups (n = 12 in each group, Fig. 4c). Reverse transcription-PCR (RT-PCR) showed that marker genes were expressed in multiple segments of the HD-2-derived nephrons, including podocytes and proximal and distal tubules, as in the HC-4-derived nephrons (Fig. 4d). To eliminate the possibility that iPSC-derived nephrons were contaminated with mouse spinal cord tissue, we performed additional RT-PCR assays, using mouse spinal cord (Sp) as a negative control (Fig. 4d). Next, to quantify the efficiency of nephron formation between HC and HD groups, we performed qRT-PCR for representative nephron markers: NPHS1 and NPHS2 as terminally differentiated podocyte-specific markers; low density lipoprotein-related protein 2 (*LRP2*) as proximal tubule marker; solute carrier family 12 member 1 (*SLC12A1*) as loop of Henle marker; polycystin 1 (*PKD*1) and polycystin 2 (*PKD2*) as distal tubule markers. In addition, we examined the expression of vascular endothelial growth factor A (*VEGFA*), which is secreted from developing podocytes and plays a critical role in nephrogenesis and growth of glomerular capillaries^33^. We obtained comparable transcriptional profiles in HC- and HD-iPSC-derived nephrons (n = 12 in each group, Fig. 4e). Histological analysis of periodic acid-Schiff (PAS) stained HD-iPSC-derived nephrons showed the presence of numerous glomeruli (Fig. 4f,g). Transmission electron microscopy revealed cell clusters that had primary foot processes (Fig. 4h). Immunostaining showed that they expressed the typical podocyte marker, WT1, in their nuclei, whereas Nephrin and Podocin were expressed on the basal sides of the podocytes (Fig. 4i–m), indicating mature podocyte formation. Some tubules expressed markers specific for proximal tubules, such as Jagged1, Lotus tetragonolobus lectin (LTL) and Megalin (Fig. 4n–p). Other tubules had macula densa-like structures (Fig. 4g) and expressed PAX2 and E-cadherin, indicative of distal tubule formation (Fig. 4q).

### HD-iPSC-derived glomeruli showed possibility to attract blood vessels similar to HC-iPSC-derived glomeruli

**Figure 5:**
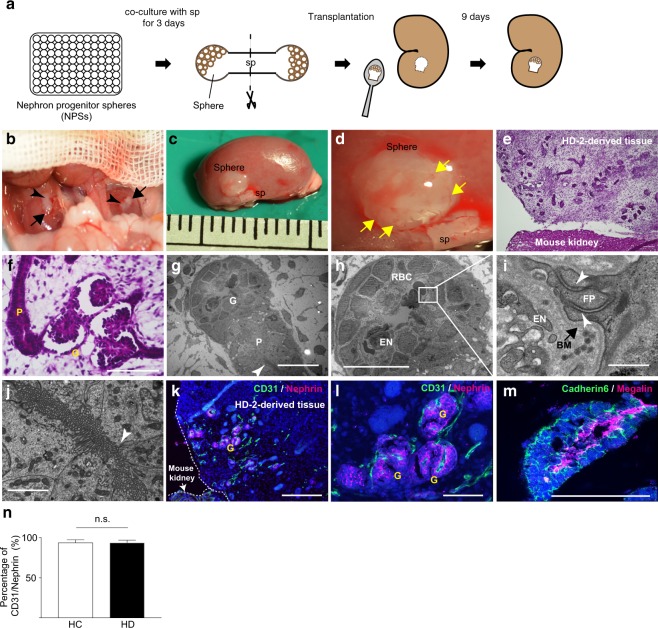
Angiogenic function of HD-iPSC-derived glomeruli compared with that of HC-iPSC-derived glomeruli. (**a**) Schematic of the transplantation method of iPSC-derived tissue beneath kidney capsules of immunodeficient mice. (**b**) HD-2-derived spheres (black arrows) and mouse embryonic spinal cords (black arrowheads) were transplanted beneath kidney capsules. (**c**) Nine days after transplantation, the size of the implanted tissues increased. sp, spinal cord. (**d**) Vascularisation of the transplanted tissue (yellow allows). (**e**,**f**) HE staining of transplanted kidney sections. G, glomerulus; P, proximal tubule. Scale bars, 100 µm. (**g**,**h**) Transmission electron microscopy of HD-2-derived tissue showing glomeruli containing red blood cells (RBC) and proximal tubule with brush border (white arrow head). EN, fenestrated endothelial cells. Scale bars, 10 µm. (**i**) Three-layer structure of the glomerular capillary wall in HD-2-derived glomeruli: fenestrated endothelial cells (EN), glomerular basement membrane (BM) and podocytes with slit diaphragm-like structures (white arrow head) between foot processes (FP). Scale bar, 0.5 µm (**j**) HD-2-derived proximal tubules having a microvillus-rich brush border at the apical side. White arrow-head, tight junction. Scale bar, 2 µm. (**k**,**l**) Immunostaining of HD-2-derived tissue showing CD31-positive endothelial cells integrated beneath Nephrin-positive structures. Scale bar, 100 µm (**k**), 50 µm (**l**) (**m**) Cadherin6-positive proximal tubule with Megalin-positive apical region. Scale bar, 50 µm. (**n**) Comparison of the percentage of CD31/Nephrin in HC- and HD-iPSC-derived glomeruli. (n = 31 and 32 respectively) HC, healthy controls; HD, haemodialysis. Data are the mean ± SEM (two-tailed, unpaired t-test).

Finally, we examined the angiogenic function of HC- and HD-iPSC-derived glomeruli *in vivo* using the cluster of differentiation 31 (CD31)/nephrin assay described by Sharmin and colleagues^34^. The authors transplanted iPSC-derived spheres, which had been co-cultured with spinal cords for one day, to initiate tubulogenesis beneath the kidney capsules of immunodeficient mice. Because the spheres might differ in their differentiation ability, it is important, before transplantation, to identify the spheres that will likely differentiate well *in vivo*. The longer spheres and spinal cords are co-cultured, the easier it is to identify the spheres that will differentiate well because of their morphological changes. Meanwhile, upon extensive co-culture, the spheres miss the time window for angiogenesis. In kidney development, the infiltration of angioblasts starts at the S-shaped body stage^35^. Resembled S-shaped bodies were observed four days after initiation of co-culture for differentiation^6,34^. Hence, we decided to co-culture NPSs derived from each iPSC line with spinal cord for three days instead of one. Then, we transplanted HC- and HD-iPSC-derived tissues beneath the kidney capsules of immunodeficient mice (Fig. 5a,b). Sharmin *et al*.^34^ found that blood vessels integrated into iPSC-derived glomeruli ten days after transplantation (11 days after initiation of co-culture). Hence, we collected iPSC-derived tissues nine days after transplantation (12 days after initiation of co-culture) and found that the size of the transplanted HD-iPSC-derived tissues had increased and small vessels had integrated from mice kidneys, similar to the transplanted HC-iPSC-derived tissues (Fig. 5c,d). Haematoxylin/eosin (HE) staining of HD-iPSC-derived tissue showed more mature glomeruli, which had open lumens in the glomerular capillary loops and proximal tubules in the transplanted tissues than in the HD-iPSC-derived nephrons cultured *in vitro* (Fig. 5e,f). Electron microscopy of HD-iPSC-derived tissue showed that glomeruli containing red blood cells had the typical three-layer structure of the glomerular capillary wall: fenestrated endothelial cells with diaphragms, glomerular basement membrane (BM) and podocytes (Fig. 5g–i). Slit diaphragm-like structures, located above the BM, were formed among podocytes. Moreover, the lumens of the proximal tubules were lined with brush borders (Fig. 5j). Immunohistochemistry of HD-iPSC-derived tissue showed that CD31-positive endothelial cells were integrated just beneath the Nephrin-positive slit diaphragm-like structures (Fig. 5k,l). The apical regions of the Cadherin6-positive proximal tubules were Megalin-positive (Fig. 5m). We used the number of CD31-positive glomeruli in the Nephrin-positive glomeruli to assess the angiogenic function *in vivo* (CD31/Nephrin assay). The percentage of CD31-positive glomeruli was over 90% in both HC- and HD-iPSC-derived glomeruli (n = 31 and 32 in the HC and HD group, respectively) and there was no significant difference between the two groups (Fig. 5n).

## Discussion

In the present study, we described the generation and characterisation of iPSC lines representing age- and sex-matched patients on haemodialysis, as well as control individuals. HD-iPSCs could differentiate into NPCs as efficiently as HC-iPSCs. HD-iPSC-derived NPCs and HC-iPSC-derived NPCs showed comparable levels of NPC marker expression. Moreover, HD-iPSC-derived NPCs showed possibility to become mature and vascularised nephrons *in vivo*, similar to HC-iPSC-derived NPCs. These findings suggest that HD-iPSCs possess sufficient nephron differentiation ability and HD-iPSC-derived nephrons can become functional *in vivo*. Therefore, our results suggest that HD-iPSCs are a useful cell source for kidney regeneration.

Several groups have succeeded in differentiating iPSCs into kidney organoids, which are 3D multicellular tissues mimicking^6–8^. To achieve kidney regeneration, we plan to use the ‘organogenic niche method’, which requires iPSC-derived NPCs as cell source for transplantation. Although Morizane *et al*.^7^ generated NPCs from iPSCs more efficiently than Taguchi *et al*.^6^, the NPCs generated with the protocol used by Taguchi *et al*.^10^ could interact with UB and glomeruli differentiated from the NPCs attract blood vessels *in vivo*^34^: both functions are indispensable in the generation of functional kidneys composed of iPSC-derived nephrons. Therefore, in this study, we used the protocol developed by Taguchi and colleagues.

Although the induction efficiency of iPSCs varied among different individuals, all iPSC lines that met the authenticity criteria for iPSCs showed almost comparable characteristics including proliferation rate (Fig. 1h,i and Supplementary Figure S1) and could differentiate into NPCs and nephrons regardless of HC or HD group upon appropriate stimulation (Figs 2d,f,i and 4e). In this study, we used 7 iPSC lines that were generated from PBMCs and 1 iPSC line that was generated from fibroblast. Although it was difficult to statistically compare the differentiation ability between iPSCs derived from PBMCs and from fibroblast, our result indicated the usefulness of HD-iPSCs derived from PBMCs in kidney regeneration. This is a very important finding for the clinical application of CKD patient-derived iPSCs because PBMCs can easily be isolated from patients, without invasive clinical procedures.

To generate kidneys from CKD-iPSC-derived NPCs, it is indispensable to identify the characteristics of HD-iPSC-derived NPCs, used as representatives of CKD-iPSC-derived NPCs, by comparing them with HC-iPSC-derived NPCs. To isolate iPSC-derived NPCs from differentiated spheres, we used an immunomagnetic separation method against NPC surface markers (ITGA8 and PDGFRA). As a result, we purified iPSC-derived NPCs (ITGA8^+^/PDGFRA^−^) efficiently and in a short time (Fig. 3d,e, Supplementary Figure S2) and showed that isolated HD-iPSC-derived NPCs expressed comparable levels of NPC markers as the HC-iPSC-derived NPCs (Fig. 3f). In kidney development, NPCs that receive signals from UB differentiate into nephrons, while UBs grow and ramify in response to signals from NPCs and connect to the distal end of nephrons^36,37^. GDNF, secreted from NPCs, is one of the NPC markers and a key molecule for reciprocal interaction. We showed that HD-iPSC-derived NPCs and HC-iPSC-derived NPCs expressed comparable levels of *GDNF*. This observation is important when generating kidneys that are composed of HD-iPSC-derived nephrons because insufficient interaction would cause retardation in UB branching, decreased kidney growth and low nephron number^38^.

Finally, we examined the angiogenic function of the iPSC-derived glomeruli. Although there are protocols for generating iPSC-derived glomeruli containing iPSC-derived endothelial cells^8,9^, glomeruli generated using the protocol we followed have been shown not to have internal vascular endothelial cells *in vitro*^34^. Nevertheless, iPSC-derived glomeruli still need to attract external endothelial cells, regardless of containing or not containing internal endothelial cells *in vivo*, that will be constituent elements of the glomerular tuft to become functional nephrons as the preexisting internal endothelial cells are lost in *in vitro* conditions^39^. In such conditions, angiogenesis, induced by the VEGFA secreted from the developing podocytes, is crucial for glomerular development and vascularisation^33,40,41^. We showed that the level of *VEGFA* expressed by HD-iPSC-derived nephrons, including developing podocytes, was comparable to that by HC-iPSC-derived nephrons *in vitro* (Fig. 4e). The result that HD-iPSC-derived glomeruli showed possibility to attract CD31-positive endothelial cells as the HC-iPSC-derived glomeruli *in vivo* without any supporting growth factors was consistent with the *in vitro* experiment (Fig. 5n). Moreover, similar to transplanted kidney organoids generated by other protocols^39,42^, transplanted HD-iPSC-derived glomerular maturation was observed with BM, fenestrated endothelium and mature podocytes in glomerular tufts. HD-iPSC-derived proximal tubular epithelial maturation was also observed with the development of brush border on the apical side. Our results suggested that HD-iPSC-derived nephrons had possibility to become functional nephrons *in vivo*, similar to the HC-iPSC-derived nephrons.

In summary, our data imply that CKD does not affect the characteristics and differentiation ability of HD-iPSCs and HD-iPSC-derived NPCs and angiogenic function of HD-iPSC-derived glomeruli. Therefore, HD-iPSCs are a plausible cell source for kidney regeneration. In addition, we found an easy and efficient method for isolating NPCs from differentiated spheres in this study. This method enables us to eliminate undifferentiated or unwanted cell population that might cause adverse effects, such as tumorigenesis, without affecting the differentiation of isolated NPCs into nephrons. In the future, we plan to use isolated HD-iPSC-derived NPCs as a cell source for the ‘organogenic niche method’. In addition, by combing this method with our previously developed urine drainage system^43^, we plan to drain urine from neo-kidneys composed of HD-iPSC-derived neo-nephrons.

However, several limitations of this study should be acknowledged. First, when we generated HD-iPSCs from PBMCs, we did not assess whether the parental PBMCs, from which iPSCs were generated, were actually affected by CKD. The longer patients suffer from CKD, more PBMCs are known to be affected by CKD^44^. Because we generated iPSCs from patients with long-standing CKD, we considered the PBMCs derived from these to be affected by CKD. Second, we could not analyse the function of iPSC-derived nephrons over a longer period because the mouse embryonic spinal cords that we used as inducers of differentiation became degenerative in a short time and the kidney capsule in which we transplanted the iPSC-derived spheres was not a suitable place for kidney development. As CKD is known to cause premature aging of PBMCs^28^, we need to assess if the characteristics of HD-iPSC-derived nephrons will be different from those of HC-iPSC-derived nephrons on long-term maintenance. Further studies are necessary to overcome this limitation; a possibility could be to provide a suitable environment, which enables long-term maintenance of iPSC-derived nephrons, mimicking the *in vivo* nephrogenic area, such as the transplantation of iPSC-derived NPCs into metanephros in which native NPCs are eliminated.

To our knowledge, this is the first report examining the potential of HD-iPSCs for kidney regeneration and their capability to generate nephrons and vascularised glomeruli from HD-iPSCs. Our data suggest that HD-iPSCs are a suitable source for kidney regeneration and indicate the usefulness of CKD-iPSCs. This study paves the way for patient-stem cell-derived kidney regeneration. Indeed, the generation of CKD patient-stem cell-derived kidneys would overcome not only organ shortages but also immune rejection issues and the life-long immunosuppression related to kidney transplantation.

## Methods

### Generation of iPSCs

Two HD-iPSC lines, HD-1 and HD-4 and three HC-iPSC lines, HC-1, HC-2 and HC-3, were generated from human PBMCs using episomal vectors, according to the protocol of the Centre for iPS Cell Research and Application (Kyoto, Japan). Two iPSC lines, HD-2 and HD-3, were generated from human PBMCs using the SeVdp vector expressing the four reprogramming factors *OCT3/4*, *SOX2*, *cMYC* and *KLF4* and removing the vector genome by RNA interference, as previously described^45,46^.

### Nephron progenitor cells/nephron induction from iPSCs

The eight iPSC lines were induced to NPSs including NPCs by a previously established method^6^. To differentiate nephrons, NPSs were co-cultured with mouse embryonic spinal cords taken from E12.5 embryos at the air-fluid interface of a polycarbonate filter (0.8 mm; Corning Inc., Corning, NY, USA) for nine days. The induction was performed at least two times for each iPSC line, independently. The percentage of the well-differentiated part per spheres was calculated using ImageJ software (National Institute of Health [NIH], Bethesda, MD, USA).

### Flow cytometric analyses

rBC2LCN-FITC (180-02991; Wako), biotinylated anti-ITGA8 (BAF4076; R&D Systems, Minneapolis, MN, USA), allophycocyanin-conjugated streptavidin (405207; BioLegend, San Diego, CA, USA) and phycoerythrin-conjugated anti-PDGFRA (323506; BioLegend) were used for cell staining. Data were acquired and analysed using the MACSQuantify analysis software (Miltenyi Biotec, Bergisch Gladbach, Germany).

### Cell sorting using the immunomagnetic cell separation system

PDGFRA negative selection was performed using Anti-PE MicroBeads UltraPure (130-105-639; Miltenyi Biotec), LS Columns (130-042-041; Miltenyi Biotec) and a MidiMACS Separator (130-042-302; Miltenyi Biotec). Then, ITGA8 positive selection was performed using Anti-Biotin MicroBeads UltraPure (130-105-637; Miltenyi Biotec), MS Columns (130-0042-201; Miltenyi Biotec) and MiniMACS Separator (130-042-102; Miltenyi Biotec). To assess the differentiation ability of the sorted cells, 3 × 10^5^ ITGA8−/PDGFRA- and ITGA8+/PDGFRA- cells were aggregated in differentiation medium with 1 µM CHIR and 5 ng/mL human FGF9 overnight at 37 °C and then co-cultured with mouse embryonic spinal cords for nine days.

### Transplantation of iPSC-derived tissues into immunodeficient mice

We modified the transplantation method previously described^34^. iPSC-derived NPSs were cultured with mouse embryonic spinal cords on polycarbonate filters for three days to initiate tubulogenesis. Before transplantation, spheres with spinal cords were cut into half and then the tissues were transplanted beneath the kidney capsules using small dispensing spoons under three types of mixed anaesthetic agents: medetomidine (Zenoaq, Fukushima, Japan), butorphanol (Meiji Seika, Tokyo, Japan) and midazolam (Astellas Pharma Inc., Tokyo, Japan). NOD/Shi-scid, IL-2RγKO Jic mice (male, 6 months old, *In-Vivo* Science Inc., Tokyo, Japan) were used as the host animals. Nine days after transplantation, the transplanted tissues were collected.

### Statistical analyses

Data are expressed as the mean ± SEM. Means were compared using the unpaired two-tailed Student’s t-test. All analyses were performed using the PRISM7 software (GraphPad Software, La Jolla, CA, USA). P < 0.05 was considered as statistically significant.

### Ethical Considerations

All animal experiments were approved by the Experimental Animal Committee of The Jikei University, Japan (Permit Number: 2016-001) and conducted in conformity with the NIH Guide for the Care and Use of Laboratory Animals. All experiments using human iPSCs were performed in accordance with institutional guidelines and approved by the Ethics Committee of The Jikei University School of Medicine (Permit Number: 25-015). All donors provided written informed consent for collection of samples and subsequent analyses.

## Electronic supplementary material

Supplementary Information

## Data Availability

The datasets generated during the current study are available from the corresponding author on reasonable request.
