# Supplementary material for: Regenerative potential of induced pluripotent stem cells derived from patients undergoing haemodialysis in kidney regeneration
Source: Sci Rep. 2018 Oct 8;8:14919. doi: 10.1038/s41598-018-33256-7 (PMC6175865; doi:10.1038/s41598-018-33256-7)
Supplement: Supplementary file 1 — Supplementary Information [file 41598_2018_33256_MOESM1_ESM.pdf]

Supplementary materials and methods

Regenerative potential of induced pluripotent stem cells derived from patients undergoing haemodialysis in kidney regeneration

Susumu Tajiri[1,4], Shuichiro Yamanaka[1], Toshinari Fujimoto[1,4], Kei Matsumoto[1], Atsuhiro Taguchi[2,3], Ryuichi Nishinakamura[2], Hirotaka James Okano[4] & Takashi Yokoo[1*]

[1]Division of Nephrology and Hypertension, Department of Internal Medicine, The Jikei University School of Medicine, 3-25-8, Nishi-Shimbashi, Minato-ku, Tokyo, 105-8461, Japan.
[2]Department of Kidney Development, Institute of Molecular Embryology and Genetics, Kumamoto University, 2-2-1, Honjo, Chuo-ku, Kumamoto, 860-0811, Japan.
[3]Department of Genome Regulation, Max Planck Institute for Molecular Genetics, Ihnestraße 63-73, 14195 Berlin, Germany.
[4]Division of Regenerative Medicine, The Jikei University School of Medicine, 3-25-8, Nishi-Shimbashi, Minato-ku, Tokyo, 105-8461, Japan.

Immunostaining, teratoma formation and karyotyping of iPSCs

The seven iPSC lines HD-1, HD-2, HD-3, HD-4, HC-1, HC-2, and HC-3, were analysed, as previously described[1].

Human iPSC Culture

Seven iPSC lines, HD-1, HD-2, HD-3, HD-4, HC-1, HC-2, and HC-3, generated in our laboratory, and HC-4 obtained from the RIKEN BioResource Centre (Ibaraki, Japan) were used in this study. The iPSC lines were maintained on MEF feeder cells, as previously described[1] and were transitioned, except HC-4, before passage 20 to feeder-free conditions on iMatrix-511 (892012; Nippi, Tokyo, Japan) in Stemfit AK02N (RCAK02N; Ajinomoto, Tokyo, Japan) for differentiation. HC-4 before passage 40 was transitioned to feeder-free conditions using otherwise identical culture conditions. To calculate the doubling time, we plated 2.0×10^4 cells into iMatrix-511-coated 12-well plates and counted the cell number over time.

RT-PCR and qRT-PCR

RNA was isolated using RNeasy Plus Micro Kit (74034; QIAGEN, Hilden, Germany) and then reverse-transcribed with random primers and oligo dT primers using the PrimeScript RT Reagent Kit with gDNA Eraser (RR047A; Takara Bio, Shiga, Japan) following the manufacturer’s instructions; the primers used for RT-PCR are listed in Supplementary Table 1.

For qRT-PCR, TaqMan gene expression assays were performed using predesigned probes that are listed in Supplementary Table 2, all from Thermo Fisher Scientific (Waltham, MA, USA). qRT-PCR was performed using an ABI 7300 real-time PCR system (Applied Biosystems, Carlsbad, CA, USA) and TaqMan Fast Advanced Master Mix (4444557; Thermo Fisher Scientific). The cDNA content in each sample was calculated using the ΔΔCt method, and target gene expression was normalized to ACTB expression. The experiments were performed in duplicate and the results are expressed as the mean ± the standard error of the mean (SEM).

Immunostaining for dissociated cells derived from NPSs

Spheres were dissociated using Neuron Dissociation Solution (291-78001; Wako, Tokyo, Japan). The dissociated cells were plated on an iMatrix-511-coated chamber slide (154941; Thermo Fisher Scientific) and incubated overnight at 37 °C. Samples were fixed with 4% paraformaldehyde. After blocking for 1 h at 25 °C, the cells were incubated overnight at 4 °C with primary antibodies. After rinsing with phosphate-buffered saline (PBS), they were incubated with secondary antibodies conjugated with AlexaFluor 488 (A21202; Thermo Fisher Scientific) and 647 (A31573; Thermo Fisher Scientific) at 25 °C for 1 h. Nuclei were stained with DAPI (S36938; Thermo Fisher Scientific). The primary antibodies were as follows: rabbit anti-WT1 (OAEB01959; Aviva Systems Biology, San Diego, CA, USA), rabbit anti-PAX2 (PRB-276P; Covance, Princeton, NJ, USA), and rabbit anti-SIX2 (11562-1-AP; Proteintech, Rosemont, IL, USA). Immunofluorescence was visualized with an LSM880 confocal microscope (Carl Zeiss, Munich, Germany) or Olympus IX73 (Olympus, Tokyo, Japan). For counting the number of marker-positive cells, three microscope fields per iPSC line were randomly selected and evaluated using ImageJ software.

PAS and HE staining and immunostaining of HD-iPSC-derived nephrons

Samples were fixed in 4% formaldehyde, embedded in optimal cutting temperature (OCT) compound (Sakura Finetechnical, Tokyo, Japan), and cryo-sectioned at 8-µm thickness. PAS and HE staining were performed according to standard procedures of histological analyses. Antigen retrieval in citrate buffer was conducted before staining. After blocking for 1 h at 25°C, the sections were incubated overnight at 4°C with primary antibodies. After rinsing with PBS, the sections were incubated with secondary antibodies conjugated with AlexaFluor 488, 555 (A31572; Thermo Fisher Scientific), and 647 specific antibodies at 25 °C for 1 h. Nuclei were stained with 4',6-diamidino-2-phenylindole (DAPI). The primary antibodies were as follows: rabbit anti-WT1 (sc-192; Santa Cruz Biotechnology, Santa Cruz, CA, USA), goat anti-nephrin (sc-19000; Santa Cruz Biotechnology), rabbit anti-podocin(ab-50339; Abcam, Cambridge, UK), rabbit anti-PAX2, fluorescein anti-LTL (FL1321; Vector Laboratories, CA, USA), mouse anti-E-cadherin (610181; BD Biosciences, CA, USA), rabbit anti-

cadherin6 (HPA007456; Sigma-Aldrich, MO, USA), goat anti-megalin (sc-16476; Santa Cruz Biotechnology), goat anti-jagged1 (sc-6011; Santa Cruz Biotechnology), and rabbit anti-CD31 (ab-28364; Abcam). Immunofluorescence was visualized with an LSM880/LSM510 confocal microscope or Olympus IX73.

Electron microscopy analyses

Samples were fixed by immersion in 2% glutaraldehyde in 0.1 M phosphate buffer (pH 7.3) at 4 °C for 30 min and post-fixed in 1% osmium tetroxide in the same buffer at 4 °C for 30 min. After dehydration in ethanol, samples were embedded in Epok 812 (Oken, Tokyo, Japan). Ultrathin sections were cut with a diamond knife, stained with uranyl acetate and lead citrate, and observed under a H-7500 electron microscope (Hitachi, Tokyo, Japan) at 80 kV.

References

1 Itoh, M., Kawagoe, S., Okano, H. J. & Nakagawa, H. Integration-free T cell-derived human induced pluripotent stem cells (iPSCs) from a patient with lymphedema-distichiasis syndrome (LDS) carrying an insertion-deletion complex mutation in the FOXC2 gene. *Stem Cell Res* **16**, 611-613, (2016).

rBC2LCN^{+} fractions			
HC-1	99.9 %	HD-1	99.7 %
HC-2	99.8 %	HD-2	99.9 %
HC-3	97.7 %	HD-3	99.6 %
HC-4	98.5 %	HD-4	99.9 %
Mean	98.9 %	Mean	99.8 %
SEM	0.6 %	SEM	0.1 %

Supplementary Figure S1. Percentage of rBC2LCN^{+} populations in HC- and HD-iPSC lines.

	HC-iPSC lines			
	ITGA8⁺/PDGFRA⁻ fractions		ITGA8⁻/PDGFRA⁻ fractions	
	Pre sorting	Post sorting	Pre sorting	Post sorting
HC-1	14.3 %	83.3 %	85.4 %	80.3 %
HC-2	23.4 %	87.3 %	76.3 %	93.6 %
HC-3	28.3 %	76.8 %	71.4 %	96.0 %
HC-4	27.7 %	89.6 %	71.6 %	96.6 %
Mean	23.4%	84.3 %	76.2 %	91.6 %
SEM	3.2 %	2.8 %	3.3 %	3.8 %

	HD-iPSC lines			
	ITGA8⁺/PDGFRA⁻ fractions		ITGA8⁻/PDGFRA⁻ fractions	
	Pre sorting	Post sorting	Pre sorting	Post sorting
HD-1	39.8 %	84.6 %	60.1 %	89.2 %
HD-2	16.9 %	83.4 %	82.9 %	85.3 %
HD-3	26.3 %	91.8 %	73.4 %	94.5 %
HD-4	19.3 %	88.9 %	80.4 %	96.6 %
Mean	25.6 %	87.2 %	74.2 %	91.4 %
SEM	5.2%	1.9 %	5.1 %	2.6 %

Supplementary Figure S2. Percentage of $ITGA8^{+}/PDGFRA^{-}$ and $ITGA8^{-}/PDGFRA^{-}$ populations in HC- and HD-iPSC-derived NPSs, pre and post sorting.

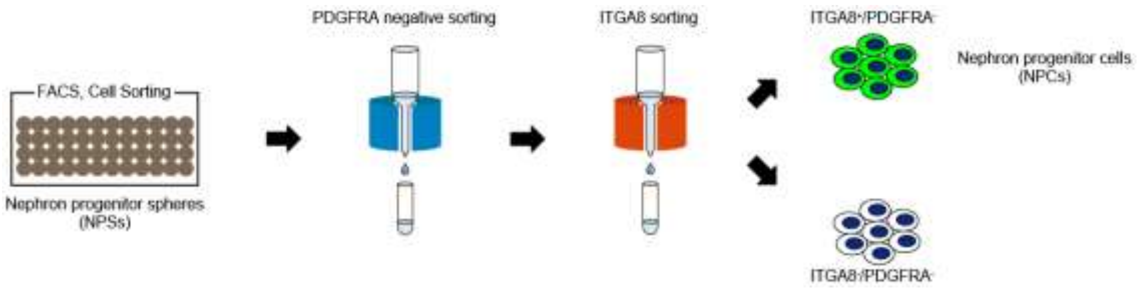

Supplementary Figure S3. Schematic of the strategy to examine the induction efficiency of the NPCs in NPSs.

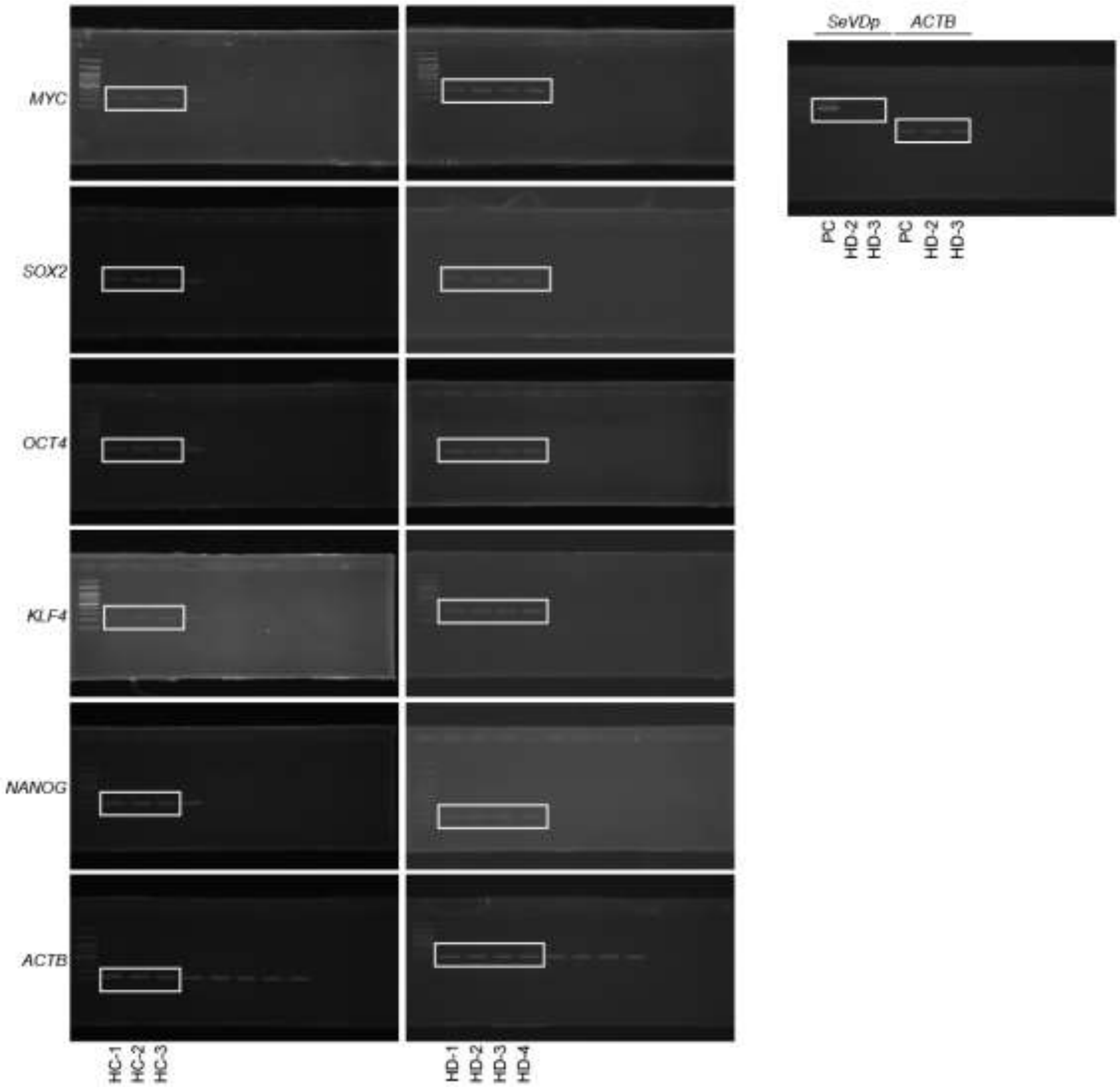

Supplementary Figure S4. Full-length gel and blots presented in Figure 1.

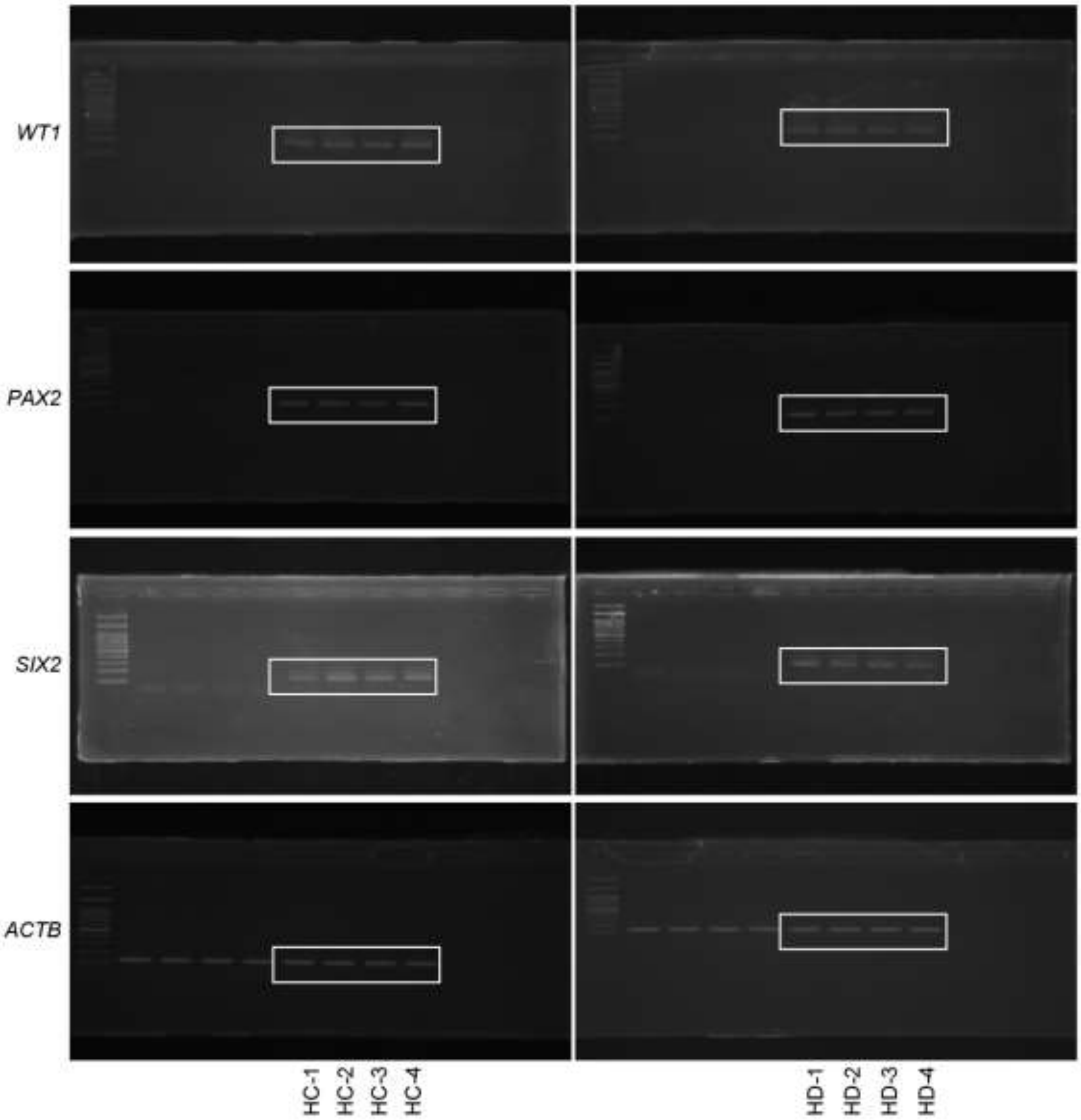

Supplementary Figure S5. Full-length gel and blots presented in Figure 2.

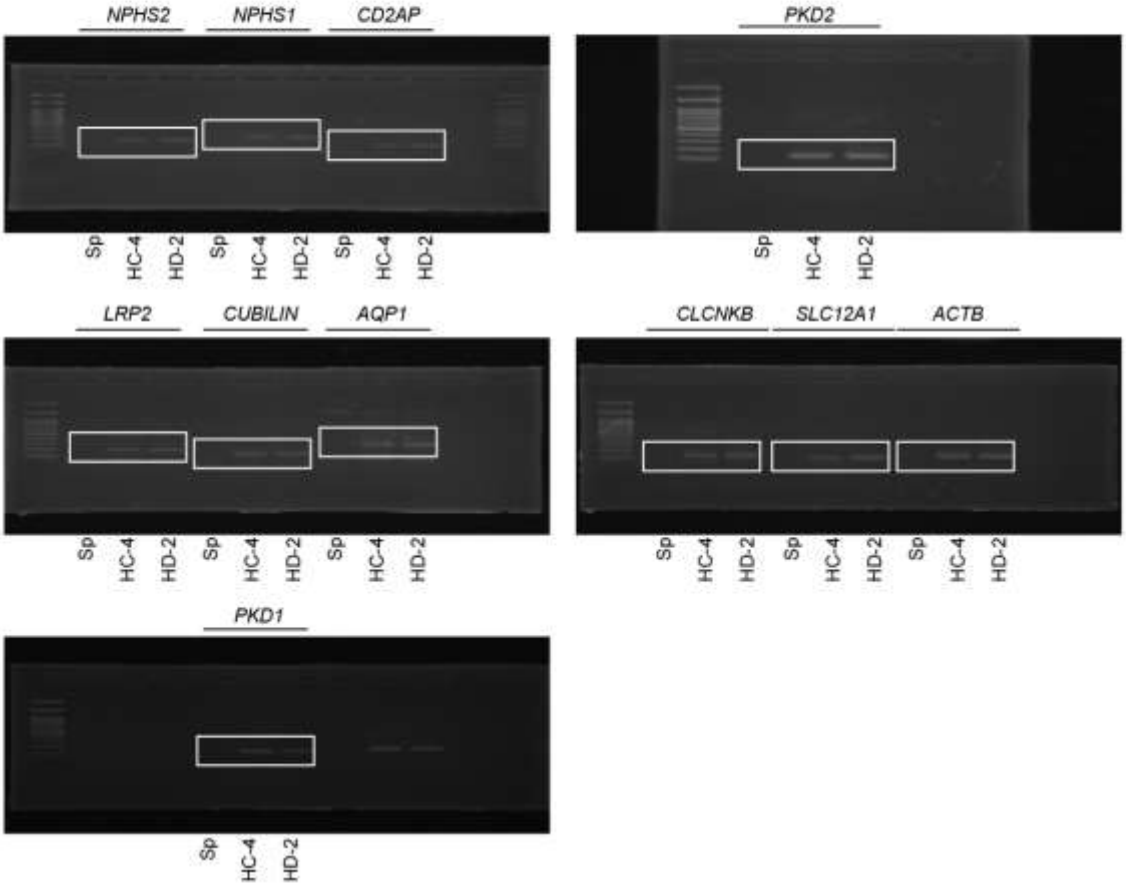

Supplementary Figure S6. Full-length gel and blots presented in Figure 4.

Supplementary Table 1. Primer sequences used for RT-PCR

	F	R
NPHS1	CAACTGGGAGAGACTGGGAGAA	CTGACAACAAGACGGAGCAG
NPHS2	GCAACAGAACTCAGGGAAGC	CCCTCAGGCATGTGACTTTT
CD2AP	AGCTCCAAAGCCTGAACTGA	ACTTGTGGAGCTGCTGGTTT
AQP1	GCCGTGACCTTGGTGGCTCA	TGGCCGCTGGTCCACACCTT
CUBILIN	ATGATGGGCCTAGCATTCAC	AAGGAATCCCTTCCCTGAGA
LRP2	AGCCTCTGGAGTTGGACAGA	ACAGTGCGGTTAGACCCATC
SLC12A1	GATGCCGTTCCCAAGATAGA	ATTCTTTGCGAGTTGCTCGT
CLCNKB	GAACCACAGCATCACCACAC	CACTATGCCCACCAGGATCT
PKD2	GTTCCACGAATACGGCAACT	CCAAAGGGAGCCCTATCTTC
PKD1	AACAGGTCTTTGGCCATCAC	TACTCGTTCAGCACGGTGAC
ACTB	CAATGTGGCCGAGGACTTTG	CATTCTCCTTAGAGAGAAGTGG

Supplementary Table 2. Primer list used for qRT-PCR

T	Hs00610080_m1
OSR1	Hs01586544_m1
WT1	Hs01103751_m1
PAX2	Hs01057416_m1
SIX2	Hs00232731_m1
GDNF	Hs01931883_s1
NPHS1	Hs00190446_m1
NPHS2	Hs00387817_m1
VEGFA	Hs00900055_m1
LRP2	Hs00189742_m1
SLC12A1	Hs00165731_m1
PKD1	Hs00947377_m1
PKD2	Hs00960946_m1
ACTB	Hs99999903_m1
